# Autism Spectrum Disorders in the Stockholm Youth Cohort: Design, Prevalence and Validity

**DOI:** 10.1371/journal.pone.0041280

**Published:** 2012-07-20

**Authors:** Selma Idring, Dheeraj Rai, Henrik Dal, Christina Dalman, Harald Sturm, Eric Zander, Brian K. Lee, Eva Serlachius, Cecilia Magnusson

**Affiliations:** 1 Division of Public Health Epidemiology, Department of Public Health Sciences, Karolinska Institutet, Stockholm, Sweden; 2 Neuropsychiatric Resource Team Southeast Stockholm, Stockholm County Council, Stockholm, Sweden; 3 Academic Unit of Psychiatry, School of Social and Community Medicine, University of Bristol, Bristol, United Kingdom; 4 Avon and Wiltshire Partnership NHS Mental Health Trust, Bristol, United Kingdom; 5 Center of Neurodevelopmental Disorders at Karolinska Institutet (KIND), Karolinska Institutet, Stockholm, Sweden; 6 Department of Women's and Children's Health, Karolinska Institutet, Stockholm, Sweden; 7 Department of Epidemiology and Biostatistics, Drexel University School of Public Health, Philadelphia, Pennsylvania, United States of America; 8 Division of Psychiatry, Department of Clinical Neuroscience, Karolinska Institutet, Stockholm, Sweden; The George Washington University, United States of America

## Abstract

**Objective:**

Reports of rising prevalence of autism spectrum disorders (ASD), along with their profound personal and societal burden, emphasize the need of methodologically sound studies to explore their causes and consequences. We here present the design of a large intergenerational resource for ASD research, along with population-based prevalence estimates of ASD and their diagnostic validity.

**Method:**

The Stockholm Youth Cohort is a record-linkage study comprising all individuals aged 0–17 years, ever resident in Stockholm County in 2001–2007 (N = 589,114). ASD cases (N = 5,100) were identified using a multisource approach, involving registers covering all pathways to ASD diagnosis and care, and categorized according to co-morbid intellectual disability. Prospectively recorded information on potential determinants and consequences of ASD were retrieved from national and regional health and administrative registers. Case ascertainment was validated through case-note review, and cross validation with co-existing cases in a national twin study.

**Results:**

The 2007 year prevalence of ASD in all children and young people was 11.5 per 1,000 (95% confidence interval 11.2–11.8), with a co-morbid intellectual disability recorded in 42.6% (41.0–44.2) of cases. We found 96.0% (92.0–98.4) of reviewed case-notes being consistent with a diagnosis of ASD, and confirmed ASD in 85.2% (66.2–95.8) of affected twins.

**Conclusions:**

Findings from this contemporary study accords with recently reported prevalence estimates from Western countries at around 1%, based on valid case ascertainment. The Stockholm Youth Cohort, in light of the availability of extensive information from Sweden's registers, constitutes an important resource for ASD research. On-going work, including collection of biological samples, will enrich the study further.

## Introduction

Autism spectrum disorders (ASD) are a group of neurodevelopmental disorders characterized by qualitative impairments in social interaction, communication and restricted and stereotyped patterns of interests and behaviours. ASD are classified as pervasive developmental disorders in current classification systems, ICD-10 and DSM-IV-TR, which include autistic disorder, Asperger disorder, pervasive developmental disorders not otherwise specified, childhood disintegrative disorder, and Rett disorder [1], [2].

The apparent prevalence of autism spectrum disorders (ASD) has risen sharply over the past two decades [3], recently estimated from approximately 1% in large studies ascertaining cases from populations with identified needs or symptoms that may be associated with ASD in the US and the UK [4], [5] to 2.6% in a South Korean study actively screening for ASD in a general population sample [6]. Although changes in diagnostic practices and wider recognition may explain part of this rise, a true increase in incidence of ASD has not been ruled out [3]. Recent Scandinavian reports are inconsistent, with ASD prevalence ranging from 0.13 to 1.2% [7]. Furthermore, Scandinavian studies have frequently ascertained ASD cases via health care registries [8], [9], [10], [11]. This approach may underestimate the prevalence of ASD, since affected children require social and educational interventions more often than health care.

Although ASD are heritable disorders, environmental factors are considered increasingly important in their etiology [12], [13]. However, the causes of ASD remains poorly understood since studies are still comparatively few and often hampered by methodological limitations such as small samples and lack of prospective data on exposures or confounders.

In light of an appearingly rising prevalence along with the profound individual and societal burden of ASD, there is an urgent need of large, prospective, population-based studies exploring modifiable risk factors. The Stockholm Youth Cohort (SYC), a register-based total population study, was established as such a resource for ASD research. We here describe the design of the SYC, including completed and ongoing data collection, and present findings from studies on the validity of our case ascertainment. Furthermore, we estimate the prevalence of ASD in Stockholm County and discuss opportunities for future research.

## Methods

### Study population and design

The Stockholm Youth Cohort is a record-linkage study comprising all children aged 0 through 17 years resident in Stockholm County at any time in 2001 through 2007 (total N = 589,114), identified through the *Register of Total Population* (provided by Statistics Sweden). The primary key for register linkage was the unique personal identification number assigned to each Swedish citizen at birth or upon arrival in Sweden for immigrants [14]. These numbers are recorded in all contacts with health care, social and administrative services, enabling complete and accurate register linkage. Register linkage was conducted by Statistics Sweden, which also replaced personal identification numbers with unique SYC identification numbers to maintain individual anonymity. To maximize the possibility of being registered with a diagnosis of ASD, all children not residing within Stockholm County for at least four years were excluded (N = 144,960). Thus, the final study population for the present report includes 444,154 individuals.

### Case ascertainment

All ASD related services, including diagnosis and follow-up health, special educational and social care are provided by services run by, or contracted with the Stockholm County Council and available free of charge. Referrals for diagnostic evaluation of suspected ASD are commonly made by child healthcare centres, whose health- and developmental surveillance program engages 99.8% of all preschool children [15]. Developmental surveillance is performed by specially trained child healthcare centre nurses at regular intervals (1, 2, 6, 10–12, 18, 36, 48 and 60 months of age), with examination by a paediatrician at key ages (2, 6, 10–12 months) and in case of developmental deviation or according to need at other age intervals. Speech abilities and language comprehension are evaluated by nurses at 36 and 48 months, and examination of sight and hearing is made at 48 months. The purpose of developmental surveillance is to ensure timely discovery of developmental problems such as cerebral paresis, speech disorders, ASD, intellectual disabilities and ADHD [16]. Referrals for diagnostic evaluation of suspected ASD may also be requested by parents through general practitioners, paediatricians, child psychiatrists, speech therapists, or by schools as well as other health or social care agencies. Diagnostic evaluations are made by multi-professional teams, typically consisting of at least a psychologist and a medical doctor at child paediatric or mental health services, according to the DSM- or ICD classification system [17]. Habilitation services are offered to all children with a diagnosed ASD, and include multimodal interventions such as special education, parental education programs, school based interventions and staff training, occupational therapy, social care, or other services as relevant.

ASD case status as of December 31, 2007, was ascertained using four national and regional registers, covering all the pathways of ASD diagnosis and care in Stockholm County that we were aware of. These included (with the respective case proportion ascertained from each source in parentheses): 1) the Habilitation Register (67.7%), 2) Clinical Database for Child and Adolescent Psychiatry in Stockholm (58.3%), 3) the VAL database (44.4%), and 4) the National Patient Register (14.1%). ASD was defined as a recorded diagnosis of ICD-9 (299) or ICD-10 (F84) in 3) or 4), and DSM-IV (299) in 2), or in case of Habilitation Register, through registration as a service recipient in these specialist centers, where a formal ASD diagnosis is a pre-requisite before referral. The registers used for case ascertainment are further described in an overview of record-linkages to national and regional registers in the SYC (Table 1). Since the information of DSM-IV subcategories was not readily available in all registers, we divided children with ASD into two groups based on the presence of co-morbid intellectual disability (defined as IQ<70 and functional impairment by international and Swedish norms). Intellectual disability was ascertained as a recorded diagnosis of 317–319 or F70-79 according to ICD-9 and ICD-10, respectively, and 317–319 according to DSM-IV [1], [2], [18], and supplemented using the Habilitation Register (Table 1), which categorizes service recipients as having autism with or without intellectual disability.

**Table 1:** Overview of record-linkages to national and regional registers in the Stockholm Youth Cohort.

Register source and web site	Register (coverage period)	Summary of available information
Statistics Sweden www.scb.se	Multi-Generation Register (1932–) [57]	Swedish residents (index persons) linked to their parents. Enables identification of first-degree relatives as well as construction of more extended family structures.
	National Population and Housing census (1960–1990) [58]	Individual and household data, e.g. employment, income, housing, household size and type of household.
	Register of the Total Population (1961–) [59]	Change in marital status, change in citizenship, internal migration, immigration and emigration from/to specified countries.
	National School Registers (1973–) [52]	Subject specific school leaving grades from compulsory- (since 1988) and upper secondary school (since 1973) as well as scores on subject specific national tests in compulsory school (since 2004).
	Integrated database for Labour Market Research (1990–) [53]	Socio-demographic data, e.g. country of birth, family income, level of education and occupational status. Updated annually.
The National Board of Health and Welfare www.socialstyrelsen.se	Medical Birth Register (1973–) [51], [60]	Perinatal characteristics, e.g. anthropometric measures and APGAR score. Maternal characteristics, e.g. pre-pregnancy weight and height, gestational weight gain, diabetes and hypertension, previous illnesses, prescribed drug use and smoking. Potential complications during pregnancy and delivery are coded according to ICD 9–10.
	National Patient Register (1964–) [61]	All inpatient care (with complete national coverage since 1987), and outpatient specialist case (since 2001) health care in specialist clinics. Diagnoses are coded according to ICD 7–10.
	Cancer Register (1958–) [62]	Information on site of tumor, histological type, stage and date and basis of diagnosis. Classification and site of tumors are coded according to ICD 7–10.
	Cause of Death Register (1952–) [63], [64]	Primary and contributory causes of death and date of death. Causes of death are coded according to ICD 7–10.
	Prescribed Drug Register (2005–) [65]	Prescribed drugs according to the national substance classification system.
Stockholm County Council www.sll.se	Stockholm Adult Psychiatric Care Register (1997–)	Adult psychiatric out- and inpatient care within Stockholm County, including diagnosis and global assessment of functioning ratings (GAF). Diagnoses are coded according to DSM-IV groupings until 2004, and according to ICD 10 since 2005.
	Clinical Database for Child and Adolescent Psychiatry in Stockholm (2001–)	Child and adolescent psychiatric in – and outpatient care within Stockholm County, including diagnosis and ratings of general functioning according to the Children's Global Assessment Scale. Diagnoses are coded according to DSM-IV groupings until 2008, and according to ICD-10 since 2009.
	Habilitation Register (1997–)	Utilization of Stockholm County Habilitation Services according to type of disability (intellectual disability, pervasive developmental disorder, mobility, vision or hearing impairments).
	VAL database (1997–)	Public health care services in Stockholm County, including diagnostic (coded according to ICD 10, available since 2006) and service provider (clinic) information.
Swedish Social Insurance Agency www.fk.se	Social Insurance Registers (1994–)	Social insurance benefits including sickness absence spells (with diagnostic information according to ICD 10 since 2005), disability pension (with diagnostic information according to ICD 9–10 since 1994), occupational injury annuity, disability allowance, old age pension and parental leave.
The Swedish National Council for Crime Prevention www.bra.se	National Convictions Register (1973–) [54]	All convictions (criminal as well as civil) in Swedish lower court.

### Validation of case ascertainment

We used two validation procedures to assess our ASD case ascertainment.

In a case note validation study, we drew a random sample of 100 ASD cases without, and 100 ASD cases with, co-morbid intellectual disability. After ethical approval, we requested de-identification of these cases by Statistics Sweden, and requested their case-notes from the relevant clinical unit responsible for their care. Using this process, we were able to retrieve case-notes for 177 cases (88.5%). The remaining 23 notes were either missing or had non-responding clinical units. Case-noteswere scrutinized according to a case validation survey constructed by a child psychiatrist and neuropediatrician (H.S., S.I.) and a learning disability psychiatrist (D.R.). The survey covered documented diagnoses, age at diagnosis, evaluation procedures (according to current County Council guidelines [17], i.e. parental interviews, child observation, psychometric and diagnostic tests and interviews, medical examination, and complementary assessments), and information on any referral to health and/or community services related to ASD with or without intellectual disability. Two clinical experts, a child psychiatrist (S.I.) and a neuropediatrician reviewed these case notes. Of the 177 notes studied, 74 were assessed independently by both assessors, blinded of each other. The criteria used to determine final case status were 1) a case-note documented diagnosis of ASD with or without intellectual disability according to ICD-9, ICD-10 and DSM-IV (see above) and at least one the remaining criteria, 2) documented evidence of use of a structured diagnostic process, 3) evidence of referral to health- and/or community services related to ASD with or without intellectual disability.

We also cross validated the SYC cases against information from a national population-based study of twins (the Child and Adolescent Twin Study in Sweden- CATSS) [19]. In CATSS, ASD was assessed via parental report and a comprehensive screening interview for neuro-developmental disorders (A-TAC), which is considered a reliable and valid screening tool for ASD [20], [21]. We identified all twins co-occurring in SYC and CATSS, and estimated the proportion of SYC twins confirmed as ASD cases according to CATSS.

### Other data collection

Using national personal identity numbers assigned to all Swedish nationals, first degree relatives (adoptive and biological parents, and siblings) have been identified through the *Multigenerational Register* [22]. Additional data available in the SYC include perinatal and social characteristics, somatic and mental disorders, legal drug use, sick-leave, disability pension, education and scholastic achievements, and crime convictions. This information has been retrieved via record linkage to national and regional health data and administrative registers, for both the index population and their relatives (Table 1).

### Statistical analyses

The Clopper-Pearson method was used to calculate the 95% confidence intervals (CI) around proportions. Results by sex and age were compared using the Chi^2^ statistic, and its associated degrees of freedom used to calculate the p-value. Inter-rater reliability was calculated as a kappa (Κ) coefficient [23] of concordance of final case status (overall and by presence of co-morbid intellectual disability) between the two reviewers. IBM SPSS version 19 was used for all statistical analyses.

### Ethics statement

The study was approved by the Regional Ethical Review Board in Stockholm. In accordance with their decision and Swedish regulations, we did not obtain informed consent from participants involved in the study.

## Results

Table 2 describes the year 2007 distribution of the Stockholm Youth Cohort study population by case status, according to sex and age. Overall, 5,100 individuals with a recorded diagnosis of ASD were identified, of which 2,172 (42.6%, 95% CI 41.0–44.2) had a registered co-morbid intellectual disability.

**Table 2:** Prevalence of autism spectrum disorders with and without intellectual disability in 2007, by sex and age.

	All SYC N (%)	All ASD				ASD without intellectual disability				ASD with intellectual disability			
		N	N per 1,000	95% CI	p-value	N	N per 1,000	95% CI	p-value	N	N per 1,000	95% CI	p-value
All	444,154 (100.0)	5,100	11.5	11.2–11.8	-	2,928	6.6	6.4–6.8	-	2,172	4.9	4.7–5.1	-
Sex					<0.0001				<0.0001				<0.0001
Male	227,64 (51.3)	3,690	16.2	15.7–16.7		2,100	9.2	8.8–9.6		1,590	7.0	6.6–7.3	
Female	216,513 (48.7)	1,410	6.5	6.2–6.9		828	3.8	3.6–4.1		582	2.7	2.5–2.9	
Age (years)					<0.0001				<0.0001				<0.0001
4–6	66,571(15.0)	434	6.5	5.9–7.1		280	4.2	3.7–4.7		154	2.3	2.0–2.7	
7–12	127,428(28.7)	1,524	12.0	11.4–12.6		794	6.2	5.8–6.7		730	5.7	5.3–6.1	
13–17	125,271(28.2)	1,834	14.6	14.0–15.3		1,082	8.6	8.1–9.2		752	6.0	5.6–6.4	
18–23	124,884(28.1)	1,308	10.5	9.9–11.0		772	6.2	5.8–6.6		536	4.3	3.9–4.7	

### Age-specific prevalence of ASD with and without intellectual disability

**Figure 1:**
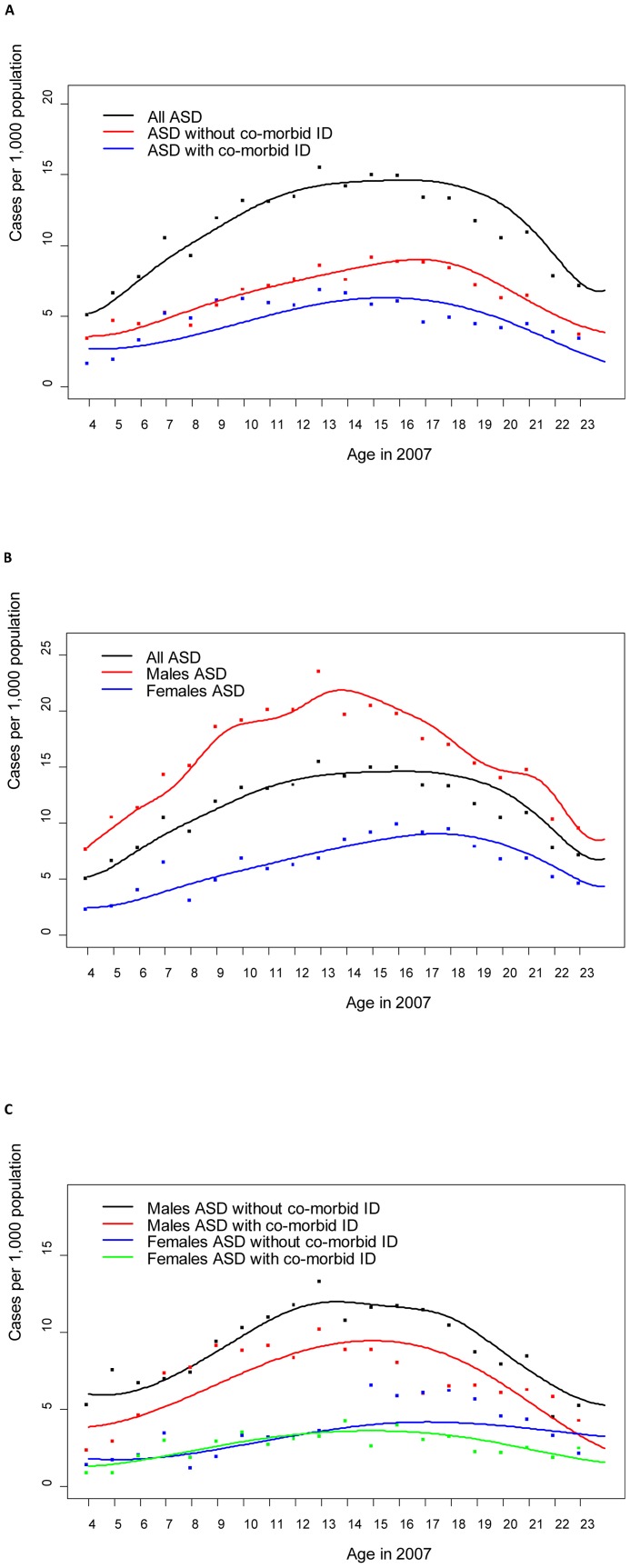
The year 2007 prevalence of autism spectrum disorders (ASD) in the Stockholm Youth Cohort. Points indicate the observed prevalence (cases per 1,000) for each age (4–23 years). An empirical mode decomposition smoothing curve is superimposed. A) ASD prevalence with or without co-morbid intellectual disability B) ASD prevalence by gender C) ASD prevalence by gender with or without co-morbid intellectual disability.

Age-specific prevalence of ASD with and without intellectual disability is presented overall and by sex in Table 2 and Figures 1a–c. The overall prevalence of ASD in 2007 was 11.5 per 1,000 (11.2–11.8), ranging from 6.5 per 1,000 among children aged 4–6 years to 14.6 per 1,000 among those aged 13–17 years. The proportion of ASD cases with co-morbid intellectual disability was 35.5% (29.4–41.6), 47.9% (45.3–50.5) and 41.0% (38.9–43.1) among children aged 4–6, 7–12 years and 13+ years, respectively. The male: female prevalence ratio for ASD overall was 2.6∶1, which was similar for ASD with and without intellectual disability (2.7∶1 versus 2.5∶1). This ratio decreased with age (from 5.1∶1 at age 8, to 1.9∶1 at age 18), especially for ASD without intellectual disability.

### Validation of case ascertainment

Of the 177 randomly selected case-notes that were scrutinized, 170 (96.0%; 92.0–98.4) were consistent with a diagnosis of ASD. Among the 7 non-confirmed ASD cases (6 boys and 1 girl), 2 presented with autistic traits, 1 had a registered diagnosis of attention/hyperactivity disorder (ADHD), 1 had a registered diagnosis of ADHD and speech and language disorder, and 1 had ADHD but had also received services at a Habilitation centre targeted at Asperger's disorder. The remaining 2 non-confirmed ASD cases had a registered diagnosis of language delay and hereditary muscle atrophy, respectively.

When assessing the validity of the cases according to their co-morbid intellectual disability status, a higher proportion of cases without (77 out of 87, 88.5%), than those with (68 out of 90, 75.6%), co-morbid intellectual disability was confirmed. Of the 22 non-confirmed ASD cases with intellectual disability, 17 had ASD but not any documented intellectual disability. Inter rater agreement on ASD status was attained in 71 of the 74 cases reviewed by both raters (corresponding to a Κ of 0.91). The proportion of confirmed cases was significantly lower in boys than in girls (p<0.05) for ASD with intellectual disability (Table 3). Children aged 7–12 years seemingly had a higher portion of confirmed cases than other age groups, but differences were not statistically significant.

**Table 3:** Confirmation of autism spectrum disorder diagnosis, with and without intellectual disability, by sex and age, according to case-notes in a random sample of 177 cases in the Stockholm Youth Cohort.

	All ASD				ASD without intellectual disability				ASD with intellectual disability			
	N	Nc (%)	95% CI	p-value	N	Nc (%)	95% CI	p-value	N	Nc (%)	95% CI	p-value
Overall	177	170 (96.0)	92.0–98.4	-	87	77 (88.5)	79.9–94.4	-	90	68 (75.6)	65.4–84.0	-
Sex				0.55				0.22				0.01
Male	121	115 (95.0)	89.5–98.2		59	50 (84.7)	73.0–92.8		62	42 (67.7)	54.7–79.1	
Female	56	55 (98.2)	90.4–100.0		28	27 (96.4)	81.6–99.9		28	26 (92.9)	76.5–99.1	
Age (years)				0.13				0.30				0.88
4–6	-	-	-									
7–12	69	65 (94.2)	85.9–98.4		28	23 (82.1)	63.1–93.9		41	32 (76.5)	62.4–89.4	
13–17	63	62 (98.4)	91.5–99.7		36	34 (94.4)	81.3–99.3		27	20 (82.1)	53.7–88.9	
18–23	45	43 (95.6)	84.9–99.5		23	20 (87.0)	66.4–97.2		22	16 (67.9)	49.8–89.3	

Nc – No. of confirmed cases.

Where such information was available (n = 148), the median age at diagnosis was 8.0 years for ASD overall (range 1–19, interquartile range [IQR] 8.0). For cases without and with intellectual disability (n = 80 and 68), the corresponding ages were 11.5 (range 4–19, IQR 6.0) and 6.0 (range 1–17, IQR 4.0) years. Girls (n = 48) were older than boys (n = 100) at diagnostic assessment (median age 11.0 as compared to 8.0 years).

In the validation sample, 54.2% of the cases were found to be diagnostically evaluated within mental health services, 35.9% within pediatric clinics and 9.8% in other settings (i.e. outside Stockholm County Council). A majority of cases were evaluated according to current regional practice guidelines [17]. All evaluations included patient history by parental report, 95.9% comprised child observation in naturalistic settings, and 92.6% included an assessment of the child's intellectual ability using standardized intelligence tests. Diagnostic evaluations were performed by multi-disciplinary teams in 95.0% of the validation sample; complementary assessments as part of the diagnostic evaluation procedure by a speech therapist were made for 38.0%, physiotherapist 19.0%, occupational therapist 14.9%, and pedagogical assessment 18.2% of cases. Assessment of intellectual ability was performed using a standardized neuropsychological evaluation based on the child's age (such as different versions of the Wechsler scales, Sniders-Oomen Nonverbal Intelligence Test and Leiter).

A total of 27 twins with of ASD were identified in SYC and, according to our cross-validation against diagnostic information in the CATSS, 23 (85.2%, 66.2–95.8) of these had an ASD confirmed in the CATSS. A total of 27 (1.0%, 0.7–1.4) of the non-case twins in our study (n = 2721) received an ASD diagnosis in CATSS.

## Discussion

In this paper the SYC is presented as a resource for population-based research on ASD. Using a multisource ascertainment in Stockholm County, 5100 cases of ASD were found in the SYC. The 2007 year prevalence of ASD in all children was 1.2%, with a recorded intellectual disability in 43% of cases. Among randomly reviewed ASD cases in a validation study, 96% were consistent with a diagnosis of ASD.

### ASD Prevalence

The 2007 year prevalence of ASD varied across age groups in the SYC, being lower amongst the oldest and youngest children. Truncation, i.e. key registers used for case ascertainment only being started in 1997 and 2001, respectively, may have deflated the observed ASD prevalence among older children. The lower rates among young children may instead be explained by reluctance among clinicians to label children with ASD already at very young ages, and/or by long diagnostic evaluation lead times [24]. This notion is supported by our finding of median age at ASD diagnosis of 8 years. This age corresponds to findings from a Danish study of a similar age group (4–26 years), reporting a mean age at ASD diagnosis of 8.9 years [25]. Despite a possible underascertainment of ASD among pre-school aged children, ASD prevalence rates in the SYC are somewhat higher compared to a recent population-based estimate of autistic disorder among a large population of 6-year olds in California largely diagnosed by 5 years of age [26], although our case definition is different. As expected, ASD cases with co-morbid intellectual disability were diagnosed at an earlier age than those without intellectual disability [27]. Further in agreement with previous findings, girls in SYC were diagnosed at a later age than boys [27], [28].

Our results are comparable to those from recent Scandinavian studies ascertaining ASD cases through active screening and diagnostic evaluation in general populations [29], [30], [31], which reported ASD prevalence estimates of 0.8–1.2%, however from small samples [29], [31]or studies possibly prone to participation bias [30]. Our results are also similar to the 1.2% and 0.9% prevalence estimates, reported by large UK and US studies, respectively, using active screening and diagnostic evaluations (through clinical assessment or case-note review) in populations with identified special education needs, symptoms associated with ASD or co-morbid conditions [4], [5]. A lower prevalence (0.6%) was, however, reported from another UK study of comparable design [32]. Studies like these may overlook affected children in the general population who, due to various barriers to care, remain unidentified [5], [32]. Indeed, studies employing active screening and diagnostic evaluations in population-based samples have found higher rates of ASD, including recent reports from the UK and South Korea where prevalence estimates approached 2% and 3%, respectively [5], [6]. These findings, however, also call for replication as estimates may be biased by non-participation [5], [6].

It is notable that cases ascertained using the National Patient Register, which predominantly covers hospital admissions, used in several epidemiological studies of ASD in Sweden [9], [10], comprised only 14% of our case sample. Similarly, case ascertainment based solely on psychiatric care may have resulted in somewhat lower prevalence estimates at 0.5% and 0.7% in the Swedish city of Gothenburg and in Denmark, respectively [8], [11].Correspondingly, more ASD cases were found in comparative age groups in the SYC than in a recent study solely ascertaining cases from specialized autism habilitation services [33] illustrating the advantage of our multiple source case ascertainment strategy.

### Sex ratio

Although the male dominance in ASD occurrence in our data is in line with one of the most consistent findings in autism, our overall male: female ratio was of a smaller magnitude than the commonly reported 4∶1 ratio [3]. Among children younger than 13 years in SYC, however, the sex ratio was in agreement with some studies [4], [5], [6], and higher than in two Japanese studies [34], [35]. Although the underlying mechanisms for the skewed sex ratio in ASD are still unknown [36], a relative under-ascertainment, and/or delayed diagnosis, of ASD in females may contribute [36], [37]. This reasoning is underpinned by our findings of a decreasing male: female ratio with increasing age, especially in ASD without intellectual disability.

### Proportion of ASD cases with Intellectual Disability

ASD is reported to presents with co-morbid intellectual disability in 25 to 70% of cases [3]. The proportion of ASD cases with intellectual disability in our study was comparable to, or somewhat lower, than that in recent studies identifying ASD through screening and diagnostic evaluations [4], [5], [29]. Kim et al, however, reported lower rates of co-morbid disability than us from their South Korean study in a general-population sample screened for ASD [6], indicating that children with ASD and normal IQ may not be identified by healthcare, special educational or social support services. The proportion of ASD cases with co-morbid intellectual disability peaked in 7–12 year olds, probably since intellectual disabilities are often diagnosed at this age [38].

### Validation of ASD cases

Through our clinical case-note review, we found 96.0% of scrutinized case-notesto be consistent with a diagnosis of ASD. Furthermore, diagnostic procedures were in accordance with practice guidelines [17] in almost all evaluations. Studies using Danish and Finnish health care registers [39], [40] have achieved similar results. Our cross-validation against the CATSS [20], [21] also generated high ASD confirmation rates (85.2%), as well as evidence that very few of the non-case twins in our study received an autism diagnosis in the CATSS. We found a higher proportion of confirmed cases with intellectual disability among girls than boys, which may be due to a later age of diagnosis in girls. The median age of diagnosis in the validation sample was relatively high considering the systematic developmental surveillance system in Sweden. There is a possibility that age at diagnosis may have been affected by previous tradition within the Swedish child- and adolescent mental health services to avoid diagnostic labelling of young children, and/or long waiting times for diagnostic evaluation, as well as its process being performed during a long time period [24]. Likewise, a high age at ASD diagnosis was found in neighbouring Denmark [25] which offers a comparable social service system, and in the UK where age at diagnosis was found to be 9.6 years in cases with Asperger's syndrome [32].

### Methodological considerations

Prevalence estimates in our study were based on ascertainment of previously diagnosed and identified cases only and may be conservative, since previous findings suggest that 40% of ASD cases may go unidentified [4], [41]. There is a possibility that young children remain unidentified in the SYC considering the relatively high age at diagnosis found in our validation study. Studies based on routine service use generally report lower ASD rates than those where active systems of screening and diagnostic evaluations were set up for research purposes [5], [42]. Nonetheless, the well-developed ASD diagnostic and care pathways in Stockholm County, including universal developmental screening at child healthcare centres, is likely to have enabled identification of a large proportion of children with at least clinically important ASD. However, since outcome misclassification cannot be ruled out, findings from this study should be interpreted with this potential bias in mind. It can be hypothesized that such misclassification is likely to be independent from exposure, and hence lead to underestimation of true associations rather than false positive findings. Since information on DSM or ICD subcategories was not readily available in all registers, we could not ASD according to these classifications. However, as boundaries between subcategories are not clearly supported in literature [43], we instead defined ASD according to DSM-V work group recommendations [43], and used an empirically supported division based on co-morbid intellectual disability [44]. Another limitation is that our case validation was based on case-note review rather than direct clinical assessment.

### Future perspectives

During recent years, a number of impressive large-scale epidemiological studies of environmental exposures, comprising biological samples have been initiated [45], [46], [47], [48], [49]. While offering important opportunities for ASD research, potential drawbacks of these studies include case ascertainment through routine health service use only [46], selection bias through non-participation [45], [47], [48], retrospective data collection [45], [48], attrition and limited generalizability to the general population [49]. Moreover, the tracking of ASD outcomes requires several years of follow-up, meaning that assembled biological samples will not yield reportable findings until years later in recently initiated prospective studies. The SYC was therefore set up to complement other endeavours, taking advantage of Sweden's rich system of total population registers containing prospectively collected data, and national biobanks containing biological samples from both neonates and pregnant women. Relatives have been identified via the Swedish Multi-Generation Register [22], and various early life determinants of ASD can be studied with control for unmeasured confounding by shared familial factors through appropriate designs such as sibling control analyses [50]. Record linkage to the Medical Birth Register [51] enables etiological research into pre- and perinatal exposures, whereas data from other health care registers with diagnostic information allows for research on morbidity and mortality outcomes in ASD. Scholastic and other social consequences of ASD can be studied through record linkage to National School Registers [52], Integrated Database for Labour Market Research [53], Social Insurance Registers and National Convictions Register [54]. In order to explore further associations on ASD and environmental exposures and confirm previous findings suggesting a potential association between ASD and hazardous air pollutants [55], there is a possibility to assign exposure levels of air pollutant concentrations to ASD cases by census tracts of birth residence. Perinatal exposure to potential neurodevelopmental toxicants [56] can also be analyzed through linkage to data on parental occupation from the 1990 census. At present, we are in the process of collecting stored biological samples for a case-control sample nested in the SYC. These include routinely collected neonatal blood spots (gathered for the purpose of screening for phenylketonuria since 1975) and maternal blood samples from antenatal health care (collected during first trisemester of pregnancy for the purpose of screening for congenital infections), stored by the Swedish Biobank. These specimens can be examined for biomarkers of relevance to ASD etiology – for example within domains of infectious agents, inflammation or steroid hormones. Lastly, means of collecting anthropometric and developmental data from existing child healthcare centre records health are currently explored.

### Conclusion

Findings from this total population cohort in Stockholm County confirm recent reports of ASD prevalence at around 1%, based on valid case ascertainment. The Stockholm Youth Cohort study design, through its exhaustive case ascertainment and subtyping of ASD by intellectual disability, provides a potent platform for further research of these complex disorders. Linkage with a large range of registers, potential for access to biological samples and developmental data opens opportunities to investigate the etiology and outcome trajectories of ASD.
